# 3-Ethyl­sulfinyl-2-(4-iodo­phen­yl)-5-methyl-1-benzofuran

**DOI:** 10.1107/S1600536810026620

**Published:** 2010-07-10

**Authors:** Hong Dae Choi, Pil Ja Seo, Byeng Wha Son, Uk Lee

**Affiliations:** a Department of Chemistry, Dongeui University, San 24 Kaya-dong Busanjin-gu, Busan 614-714, Republic of Korea; b Department of Chemistry, Pukyong National University, 599-1 Daeyeon 3-dong, Nam-gu, Busan 608-737, Republic of Korea

## Abstract

In the title compound, C_17_H_15_IO_2_S, the 4-iodo­phenyl ring makes a dihedral angle of 35.39 (8)° with the plane of the benzofuran fragment. In the crystal, mol­ecules are linked by inter­molecular C—H⋯O and C—H⋯π inter­actions, and an I⋯O contact [3.378 (2) Å]. The crystal structure also exhibits aromatic π–π inter­actions between the benzene rings of neighbouring mol­ecules [centroid–centroid distance = 3.495 (3) Å].

## Related literature

For the pharmacological activity of benzofuran compounds, see: Aslam *et al.* (2006 ▶); Galal *et al.* (2009 ▶); Khan *et al.* (2005 ▶). For natural products with benzofuran rings, see: Akgul & Anil (2003 ▶); Soekamto *et al.* (2003 ▶). For the structures of related 3-ethyl­sulfinyl-5-halo-2-(4-halophen­yl)-1-benzofuran derivatives, see: Choi *et al.* (2010**a* ▶,*b* ▶,c* ▶). For a review of halogen bonding, see: Politzer *et al.* (2007 ▶).
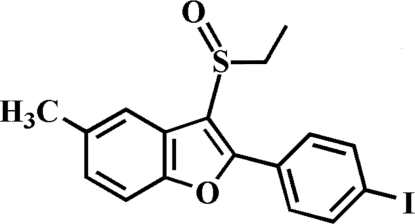


## Experimental

### 

#### Crystal data

C_17_H_15_IO_2_S
*M* *_r_* = 410.25
Monoclinic, 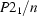

*a* = 10.1034 (3) Å
*b* = 13.1942 (4) Å
*c* = 11.9933 (3) Å
β = 92.634 (1)°
*V* = 1597.09 (8) Å^3^
*Z* = 4
Mo *K*α radiation
μ = 2.14 mm^−1^
*T* = 173 K
0.32 × 0.27 × 0.09 mm

#### Data collection

Bruker SMART APEXII CCD diffractometer
Absorption correction: multi-scan (*SADABS*; Bruker, 2009 ▶) *T*_min_ = 0.551, *T*_max_ = 0.834
15129 measured reflections
3667 independent reflections
3366 reflections with *I* > 2σ(*I*)
*R*_int_ = 0.025

#### Refinement

*R*[*F* ^2^ > 2σ(*F* ^2^)] = 0.026
*wR*(*F* ^2^) = 0.069
*S* = 1.07
3667 reflections
192 parameters
H-atom parameters constrained
Δρ_max_ = 0.96 e Å^−3^
Δρ_min_ = −0.69 e Å^−3^

### 

Data collection: *APEX2* (Bruker, 2009 ▶); cell refinement: *SAINT* (Bruker, 2009 ▶); data reduction: *SAINT*; program(s) used to solve structure: *SHELXS97* (Sheldrick, 2008 ▶); program(s) used to refine structure: *SHELXL97* (Sheldrick, 2008 ▶); molecular graphics: *ORTEP-3* (Farrugia, 1997 ▶) and *DIAMOND* (Brandenburg, 1998 ▶); software used to prepare material for publication: *SHELXL97*.

## Supplementary Material

Crystal structure: contains datablocks global, I. DOI: 10.1107/S1600536810026620/bg2357sup1.cif

Structure factors: contains datablocks I. DOI: 10.1107/S1600536810026620/bg2357Isup2.hkl

Additional supplementary materials:  crystallographic information; 3D view; checkCIF report

## Figures and Tables

**Table 1:** Hydrogen-bond geometry (Å, °) *Cg* is the centroid of the C2–C7 benzene ring.

*D*—H⋯*A*	*D*—H	H⋯*A*	*D*⋯*A*	*D*—H⋯*A*
C16—H16*B*⋯O2^i^	0.97	2.60	3.341 (3)	134
C11—H11⋯*Cg*^ii^	0.93	2.61	3.378 (3)	141

Symmetry codes: (i) 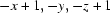; (ii) 
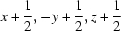
.

**Fig. 1.**
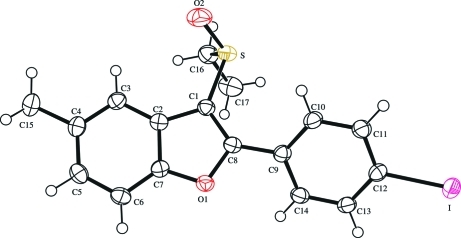
The molecular structure of the title compound with the atom numbering scheme. Displacement ellipsoids are drawn at the 50% probability level. H atoms are presented as a small spheres of arbitrary radius.

**Fig. 2.**
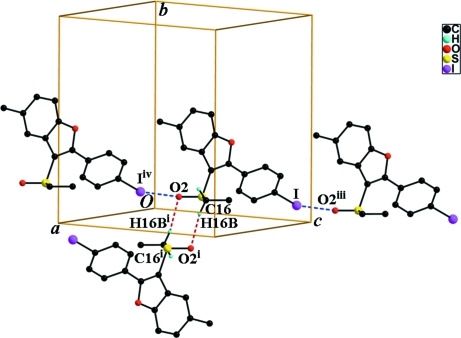
C–H···O and I···O interactions (dotted lines) in the crystal structure of the title compound. [Symmetry codes: (i) -x + 1, -y, -z + 1; (iii) x, y, z + 1; (iv) x, y, z -1 .]

**Fig. 3.**
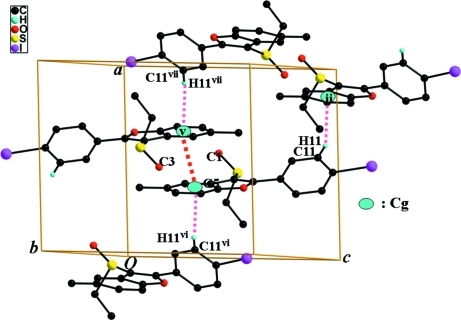
C–H···π, and π–π interactions (dotted lines) in the crystal structure of the title compound. Cg denotes the ring centroid. [Symmetry codes: (ii) x + 1/2, -y + 1/2, z + 1/2; (v) -x + 1, -y + 1, -z + 1; (vi) x - 1/2, -y + 1/2, z - 1/2; (vii) -x + 3/2, y + 1/2, -z + 3/2.]

## supplementary crystallographic information

### Comment

The benzofuran ring system show interesting pharmacological properties such as antifungal (Aslam *et al.*,  2006), antitumor and antiviral (Galal *et al.*, 2009), antimicrobial (Khan *et al.*, 2005) activities. These compounds widely occur in nature (Akgul & Anil, 2003; Soekamto *et al.*, 2003). As a part of our continuing studies of the substituent effect on the solid state structures of 3-ethylsulfinyl-5-halo-2-(4-halophenyl)-1-benzofuran analogues (Choi *et al.*, 2010*a,b,c*), we report the crystal structure of the title compound (Fig. 1).

The benzofuran unit is essentially planar, with a mean deviation of 0.021 (2) Å from the least-squares plane defined by the nine constituent atoms. The dihedral angle formed by the benzofuran plane and the 4-iodophenyl ring is 35.39 (8)°. The crystal packing (Fig. 2) is stabilized by a weak intermolecular C—H···O hydrogen bond between the methylene H atom of the ethyl group and the oxygen of the S═O unit, with a C16—16B···O2^i^ (Table 1), and by an I···O halogen-bonding between the iodine and the oxygen of the S═O unit [I···O2^iii^ = 3.378 (2) Å; C12—I···O2^iii^ = 162.46 (7)°] (Politzer *et al.*, 2007). The molecular packing (Fig. 3) is further stabilized by an intermolecular C—H···π interaction between the 4-iodophenyl H atom and the benzene ring of a neighbouring molecule, with a C11—H11···Cg^ii^ (Table 1), and by an aromatic π–π interaction between the benzene rings of neighbouring molecules, with a Cg···Cg^v^ distance of 3.495 (3) Å (Cg is the centroid of the C2–C7 benzene ring).

### Experimental

77% 3-chloroperoxybenzoic acid (202 mg, 0.9 mmol) was added in small portions to a stirred solution of 3-ethylsulfanyl-2-(4-iodophenyl)-5-methyl-1-benzofurans (315 mg, 0.8 mmol) in dichloromethane (40 mL) at 273 K. After being stirred at room temperature for 4h, the mixture was washed with saturated sodium bicarbonate solution and the organic layer was separated, dried over magnesium sulfate, filtered and concentrated at reduced pressure. The residue was purified by column chromatography (hexane-ethyl acetate, 1:2 v/v) to afford the title compound as a colorless solid [yield 79%, m.p. 431–432 K;  *R*_f_ = 0.63 (hexane–ethyl acetate, 1:2 v/v)]. Single crystals suitable for X-ray diffraction were prepared by slow evaporation of a solution of the title compound in tetrahydrofuran at room temperature.

### Refinement

All H atoms were positioned geometrically and refined using a riding model, with C—H = 0.93 Å for aryl, 0.96 Å for methylene and methyl H atoms.  *U*_iso_(H) = 1.2*U*_eq_(C) for aryl and methylene H atoms, and 1.5*U*_eq_(C) for methyl H atoms.

### Crystal data

**Table:**

C_17_H_15_IO_2_S	*F*(000) = 808
*M**_r_* = 410.25	*D*_x_ = 1.706 Mg m^−^^3^
Monoclinic, *P*2_1_/*n*	Mo *K*α radiation, λ = 0.71073 Å
Hall symbol: -P 2yn	Cell parameters from 9921 reflections
*a* = 10.1034 (3) Å	θ = 2.3–27.5°
*b* = 13.1942 (4) Å	µ = 2.14 mm^−^^1^
*c* = 11.9933 (3) Å	*T* = 173 K
β = 92.634 (1)°	Block, colourless
*V* = 1597.09 (8) Å^3^	0.32 × 0.27 × 0.09 mm
*Z* = 4	

### Data collection

**Table:**

Bruker SMART APEXII CCD diffractometer	3667 independent reflections
Radiation source: rotating anode	3366 reflections with *I* > 2σ(*I*)
graphite multilayer	*R*_int_ = 0.025
Detector resolution: 10.0 pixels mm^-1^	θ_max_ = 27.5°, θ_min_ = 2.3°
φ and ω scans	*h* = −8→13
Absorption correction: multi-scan (*SADABS*; Bruker, 2009)	*k* = −17→10
*T*_min_ = 0.551, *T*_max_ = 0.834	*l* = −15→15
15129 measured reflections	

### Refinement

**Table:**

Refinement on *F*^2^	Primary atom site location: structure-invariant direct methods
Least-squares matrix: full	Secondary atom site location: difference Fourier map
*R*[*F*^2^ > 2σ(*F*^2^)] = 0.026	Hydrogen site location: difference Fourier map
*wR*(*F*^2^) = 0.069	H-atom parameters constrained
*S* = 1.07	*w* = 1/[σ^2^(*F*_o_^2^) + (0.0328*P*)^2^ + 1.5593*P*] where *P* = (*F*_o_^2^ + 2*F*_c_^2^)/3
3667 reflections	(Δ/σ)_max_ = 0.001
192 parameters	Δρ_max_ = 0.96 e Å^−^^3^
0 restraints	Δρ_min_ = −0.69 e Å^−^^3^

### Special details

**Table:**

Geometry. All esds (except the esd in the dihedral angle between two l.s. planes) are estimated using the full covariance matrix. The cell esds are taken into account individually in the estimation of esds in distances, angles and torsion angles; correlations between esds in cell parameters are only used when they are defined by crystal symmetry. An approximate (isotropic) treatment of cell esds is used for estimating esds involving l.s. planes.
Refinement. Refinement of F^2^ against ALL reflections. The weighted R-factor wR and goodness of fit S are based on F^2^, conventional R-factors R are based on F, with F set to zero for negative F^2^. The threshold expression of F^2^ > 2sigma(F^2^) is used only for calculating R-factors(gt) etc. and is not relevant to the choice of reflections for refinement. R-factors based on F^2^ are statistically about twice as large as those based on F, and R- factors based on ALL data will be even larger.

### Fractional atomic coordinates and isotropic or equivalent isotropic displacement parameters (Å^2^)

**Table:**

	*x*	*y*	*z*	*U*_iso_*/*U*_eq_	
I	0.491732 (16)	0.129285 (13)	1.209790 (12)	0.03433 (7)	
S	0.44476 (6)	0.12571 (4)	0.57565 (5)	0.02928 (13)	
O2	0.5466 (2)	0.12728 (14)	0.48973 (19)	0.0451 (5)	
O1	0.36148 (16)	0.39156 (12)	0.71054 (13)	0.0269 (3)	
C1	0.4080 (2)	0.25304 (17)	0.60955 (18)	0.0254 (4)	
C2	0.3800 (2)	0.33516 (18)	0.53236 (19)	0.0253 (4)	
C3	0.3795 (2)	0.34768 (18)	0.41673 (19)	0.0281 (5)	
H3	0.3964	0.2931	0.3704	0.034*	
C4	0.3531 (2)	0.44313 (19)	0.3722 (2)	0.0292 (5)	
C5	0.3273 (2)	0.52457 (18)	0.4432 (2)	0.0300 (5)	
H5	0.3108	0.5881	0.4120	0.036*	
C6	0.3253 (2)	0.51387 (18)	0.5580 (2)	0.0290 (5)	
H6	0.3068	0.5680	0.6045	0.035*	
C7	0.3525 (2)	0.41812 (17)	0.59933 (18)	0.0253 (4)	
C8	0.3968 (2)	0.29066 (17)	0.71460 (18)	0.0255 (4)	
C9	0.4144 (2)	0.24931 (17)	0.82760 (18)	0.0261 (4)	
C10	0.5115 (2)	0.1770 (2)	0.85308 (19)	0.0309 (5)	
H10	0.5633	0.1525	0.7969	0.037*	
C11	0.5319 (3)	0.14090 (19)	0.9612 (2)	0.0315 (5)	
H11	0.5966	0.0923	0.9775	0.038*	
C12	0.4547 (2)	0.17826 (18)	1.04487 (18)	0.0276 (5)	
C13	0.3559 (2)	0.24893 (18)	1.02076 (19)	0.0288 (5)	
H13	0.3034	0.2725	1.0769	0.035*	
C14	0.3359 (2)	0.28432 (18)	0.91224 (19)	0.0283 (5)	
H14	0.2697	0.3317	0.8959	0.034*	
C15	0.3528 (3)	0.4598 (2)	0.2475 (2)	0.0384 (6)	
H15A	0.4124	0.4127	0.2151	0.058*	
H15B	0.3810	0.5278	0.2326	0.058*	
H15C	0.2649	0.4496	0.2156	0.058*	
C16	0.2900 (3)	0.0929 (2)	0.5052 (2)	0.0372 (6)	
H16A	0.2704	0.1419	0.4465	0.045*	
H16B	0.2984	0.0270	0.4705	0.045*	
C17	0.1766 (3)	0.0902 (2)	0.5821 (3)	0.0486 (7)	
H17A	0.1975	0.0446	0.6428	0.073*	
H17B	0.0981	0.0673	0.5417	0.073*	
H17C	0.1620	0.1569	0.6109	0.073*	

### Atomic displacement parameters (Å^2^)

**Table:**

	*U*^11^	*U*^22^	*U*^33^	*U*^12^	*U*^13^	*U*^23^
I	0.03738 (11)	0.04108 (12)	0.02435 (9)	0.00488 (7)	−0.00062 (7)	0.00094 (6)
S	0.0299 (3)	0.0259 (3)	0.0322 (3)	0.0037 (2)	0.0021 (2)	−0.0051 (2)
O2	0.0406 (11)	0.0417 (11)	0.0547 (13)	0.0043 (8)	0.0192 (10)	−0.0043 (9)
O1	0.0306 (8)	0.0241 (8)	0.0258 (8)	0.0025 (6)	−0.0021 (6)	−0.0040 (6)
C1	0.0272 (11)	0.0226 (11)	0.0264 (10)	0.0002 (8)	0.0006 (8)	−0.0023 (8)
C2	0.0228 (10)	0.0249 (11)	0.0283 (11)	−0.0021 (9)	0.0007 (8)	−0.0019 (9)
C3	0.0284 (11)	0.0286 (11)	0.0275 (11)	−0.0032 (9)	0.0035 (9)	−0.0028 (9)
C4	0.0242 (11)	0.0340 (12)	0.0294 (11)	−0.0053 (9)	0.0023 (9)	0.0032 (9)
C5	0.0259 (11)	0.0265 (11)	0.0373 (12)	−0.0018 (9)	−0.0010 (9)	0.0042 (9)
C6	0.0267 (11)	0.0257 (11)	0.0344 (12)	0.0002 (9)	−0.0014 (9)	−0.0040 (9)
C7	0.0235 (10)	0.0271 (11)	0.0253 (10)	−0.0013 (9)	−0.0010 (8)	−0.0028 (8)
C8	0.0243 (10)	0.0242 (11)	0.0278 (10)	0.0020 (8)	−0.0005 (8)	−0.0027 (8)
C9	0.0260 (11)	0.0277 (11)	0.0244 (10)	0.0001 (9)	−0.0017 (8)	−0.0032 (8)
C10	0.0283 (12)	0.0400 (14)	0.0244 (10)	0.0086 (10)	0.0020 (9)	−0.0033 (9)
C11	0.0288 (12)	0.0362 (13)	0.0290 (11)	0.0079 (10)	−0.0031 (9)	−0.0034 (9)
C12	0.0293 (11)	0.0298 (12)	0.0234 (10)	−0.0015 (9)	−0.0014 (9)	−0.0037 (9)
C13	0.0308 (12)	0.0295 (12)	0.0265 (11)	0.0034 (9)	0.0035 (9)	−0.0048 (9)
C14	0.0280 (11)	0.0258 (11)	0.0310 (11)	0.0049 (9)	−0.0002 (9)	−0.0032 (9)
C15	0.0434 (15)	0.0424 (15)	0.0298 (12)	−0.0048 (12)	0.0044 (10)	0.0068 (11)
C16	0.0397 (14)	0.0326 (13)	0.0392 (13)	−0.0019 (11)	−0.0007 (11)	−0.0048 (11)
C17	0.0383 (15)	0.0398 (15)	0.068 (2)	−0.0042 (12)	0.0118 (14)	−0.0039 (14)

### Geometric parameters (Å, °)

**Table:**

I—C12	2.098 (2)	C9—C10	1.392 (3)
I—O2^i^	3.378 (2)	C9—C14	1.395 (3)
S—O2	1.490 (2)	C10—C11	1.388 (3)
S—C1	1.772 (2)	C10—H10	0.9300
S—C16	1.796 (3)	C11—C12	1.390 (3)
O1—C7	1.378 (3)	C11—H11	0.9300
O1—C8	1.379 (3)	C12—C13	1.386 (3)
C1—C8	1.364 (3)	C13—C14	1.389 (3)
C1—C2	1.445 (3)	C13—H13	0.9300
C2—C7	1.393 (3)	C14—H14	0.9300
C2—C3	1.396 (3)	C15—H15A	0.9600
C3—C4	1.389 (3)	C15—H15B	0.9600
C3—H3	0.9300	C15—H15C	0.9600
C4—C5	1.402 (3)	C16—C17	1.503 (4)
C4—C15	1.513 (3)	C16—H16A	0.9700
C5—C6	1.386 (3)	C16—H16B	0.9700
C5—H5	0.9300	C17—H17A	0.9600
C6—C7	1.380 (3)	C17—H17B	0.9600
C6—H6	0.9300	C17—H17C	0.9600
C8—C9	1.464 (3)		
			
C12—I—O2^i^	162.46 (7)	C11—C10—H10	119.6
O2—S—C1	107.71 (11)	C9—C10—H10	119.6
O2—S—C16	107.00 (13)	C10—C11—C12	119.3 (2)
C1—S—C16	98.58 (12)	C10—C11—H11	120.4
C7—O1—C8	106.57 (17)	C12—C11—H11	120.4
C8—C1—C2	107.27 (19)	C13—C12—C11	120.7 (2)
C8—C1—S	125.75 (18)	C13—C12—I	119.95 (16)
C2—C1—S	126.94 (17)	C11—C12—I	119.34 (18)
C7—C2—C3	119.2 (2)	C12—C13—C14	119.6 (2)
C7—C2—C1	104.97 (19)	C12—C13—H13	120.2
C3—C2—C1	135.8 (2)	C14—C13—H13	120.2
C4—C3—C2	118.7 (2)	C13—C14—C9	120.5 (2)
C4—C3—H3	120.6	C13—C14—H14	119.8
C2—C3—H3	120.6	C9—C14—H14	119.8
C3—C4—C5	120.0 (2)	C4—C15—H15A	109.5
C3—C4—C15	120.3 (2)	C4—C15—H15B	109.5
C5—C4—C15	119.8 (2)	H15A—C15—H15B	109.5
C6—C5—C4	122.4 (2)	C4—C15—H15C	109.5
C6—C5—H5	118.8	H15A—C15—H15C	109.5
C4—C5—H5	118.8	H15B—C15—H15C	109.5
C7—C6—C5	116.0 (2)	C17—C16—S	112.9 (2)
C7—C6—H6	122.0	C17—C16—H16A	109.0
C5—C6—H6	122.0	S—C16—H16A	109.0
O1—C7—C6	125.7 (2)	C17—C16—H16B	109.0
O1—C7—C2	110.6 (2)	S—C16—H16B	109.0
C6—C7—C2	123.6 (2)	H16A—C16—H16B	107.8
C1—C8—O1	110.56 (19)	C16—C17—H17A	109.5
C1—C8—C9	135.1 (2)	C16—C17—H17B	109.5
O1—C8—C9	114.37 (18)	H17A—C17—H17B	109.5
C10—C9—C14	119.0 (2)	C16—C17—H17C	109.5
C10—C9—C8	120.9 (2)	H17A—C17—H17C	109.5
C14—C9—C8	120.1 (2)	H17B—C17—H17C	109.5
C11—C10—C9	120.9 (2)		
			
O2—S—C1—C8	−134.9 (2)	C2—C1—C8—O1	0.8 (3)
C16—S—C1—C8	114.1 (2)	S—C1—C8—O1	−177.22 (16)
O2—S—C1—C2	47.4 (2)	C2—C1—C8—C9	−178.6 (2)
C16—S—C1—C2	−63.6 (2)	S—C1—C8—C9	3.3 (4)
C8—C1—C2—C7	−0.4 (3)	C7—O1—C8—C1	−1.0 (2)
S—C1—C2—C7	177.68 (17)	C7—O1—C8—C9	178.56 (19)
C8—C1—C2—C3	177.1 (3)	C1—C8—C9—C10	34.9 (4)
S—C1—C2—C3	−4.8 (4)	O1—C8—C9—C10	−144.5 (2)
C7—C2—C3—C4	0.8 (3)	C1—C8—C9—C14	−146.9 (3)
C1—C2—C3—C4	−176.4 (2)	O1—C8—C9—C14	33.7 (3)
C2—C3—C4—C5	−0.2 (3)	C14—C9—C10—C11	−1.0 (4)
C2—C3—C4—C15	179.5 (2)	C8—C9—C10—C11	177.2 (2)
C3—C4—C5—C6	−0.7 (4)	C9—C10—C11—C12	−0.4 (4)
C15—C4—C5—C6	179.6 (2)	C10—C11—C12—C13	1.6 (4)
C4—C5—C6—C7	1.0 (3)	C10—C11—C12—I	−176.97 (19)
C8—O1—C7—C6	−176.9 (2)	C11—C12—C13—C14	−1.4 (4)
C8—O1—C7—C2	0.8 (2)	I—C12—C13—C14	177.17 (18)
C5—C6—C7—O1	177.0 (2)	C12—C13—C14—C9	0.0 (4)
C5—C6—C7—C2	−0.3 (3)	C10—C9—C14—C13	1.2 (4)
C3—C2—C7—O1	−178.3 (2)	C8—C9—C14—C13	−177.1 (2)
C1—C2—C7—O1	−0.3 (2)	O2—S—C16—C17	−179.0 (2)
C3—C2—C7—C6	−0.6 (4)	C1—S—C16—C17	−67.5 (2)
C1—C2—C7—C6	177.4 (2)		

Symmetry codes: (i) *x*, *y*, *z*+1.

### Hydrogen-bond geometry (Å, °)

**Table:**

Cg is the centroid of the C2–C7 benzene ring.

**Table:**

*D*—H···*A*	*D*—H	H···*A*	*D*···*A*	*D*—H···*A*
C16—H16B···O2^ii^	0.97	2.60	3.341 (3)	134
C11—H11···Cg^iii^	0.93	2.61	3.378 (3)	141

Symmetry codes: (ii) −*x*+1, −*y*, −*z*+1; (iii) *x*+1/2, −*y*+1/2, *z*+1/2.

## References

[bb1] Akgul, Y. Y. & Anil, H. (2003). *Phytochemistry*, **63**, 939–943. 10.1016/s0031-9422(03)00357-1 12895543

[bb2] Aslam, S. N., Stevenson, P. C., Phythian, S. J., Veitch, N. C. & Hall, D. R. (2006). *Tetrahedron*, **62**, 4214–4226.

[bb3] Brandenburg, K. (1998). *DIAMOND* Crystal Impact GbR, Bonn, Germany.

[bb4] Bruker (2009). *APEX2* *SADABS* and *SAINT* Bruker AXS Inc., Madison, Wisconsin, USA.

[bb5] Choi, H. D., Seo, P. J., Son, B. W. & Lee, U. (2010*a*). *Acta Cryst.* E**66**, o770. 10.1107/S1600536810008172 PMC2984045 21580614

[bb6] Choi, H. D., Seo, P. J., Son, B. W. & Lee, U. (2010*b*). *Acta Cryst.* E**66**, o1043. 10.1107/S1600536810012717 PMC2979084 21579104

[bb7] Choi, H. D., Seo, P. J., Son, B. W. & Lee, U. (2010*c*). *Acta Cryst.* E**66**, o1862. 10.1107/S1600536810024736 PMC3006759 21588059

[bb8] Farrugia, L. J. (1997). *J. Appl. Cryst.* **30**, 565.

[bb9] Galal, S. A., Abd El-All, A. S., Abdallah, M. M. & El-Diwani, H. I. (2009). *Bioorg. Med. Chem. Lett* **19**, 2420–2428. 10.1016/j.bmcl.2009.03.069 19345581

[bb10] Khan, M. W., Alam, M. J., Rashid, M. A. & Chowdhury, R. (2005). *Bioorg. Med. Chem* **13**, 4796–4805. 10.1016/j.bmc.2005.05.009 15964760

[bb11] Politzer, P., Lane, P., Concha, M. C., Ma, Y. & Murray, J. S. (2007). *J. Mol. Model* **13**, 305–311. 10.1007/s00894-006-0154-7 17013631

[bb12] Sheldrick, G. M. (2008). *Acta Crys*t. A**64**, 112–122.

[bb13] Soekamto, N. H., Achmad, S. A., Ghisalberti, E. L., Hakim, E. H. & Syah, Y. M. (2003). *Phytochemistry*, **64**, 831–834. 10.1016/j.phytochem.2003.08.009 14559276

